# Intrapersonal, social and physical environmental determinants of moderate-to-vigorous physical activity in working-age women: a systematic review protocol

**DOI:** 10.1186/2046-4053-3-132

**Published:** 2014-11-04

**Authors:** Stephanie A Prince, Jennifer L Reed, Kara A Nerenberg, Elizabeth A Kristjansson, Swapnil Hiremath, Kristi B Adamo, Heather E Tulloch, Kerri-Anne Mullen, J George Fodor, Erica Wright, Robert D Reid

**Affiliations:** 1 Division of Prevention and Rehabilitation, University of Ottawa Heart Institute, 40 Ruskin Street, Ottawa, ON K1Y 4 W7, Canada; 2 Ottawa Hospital Research Institute, 725 Parkdale Avenue, Ottawa, ON K1Y 4E9, Canada; 3 School of Psychology, University of Ottawa, 135 Jean Jacques Lussier, Ottawa, ON K1N 6 N5, Canada; 4 Division of Nephrology, The Ottawa Hospital, 1053 Carling Avenue, Ottawa, ON K1Y 4E9, Canada; 5 Healthy Active Living and Obesity Research Group, Children’s Hospital of Eastern Ontario Research Institute, 401 Smyth Road, K1H 8 L1 Ottawa, ON, Canada; 6 Berkman Library, University of Ottawa Heart Institute, 40 Ruskin Street, Ottawa, ON K1Y 4 W7, Canada

**Keywords:** Moderate-to-vigorous physical activity, Socio-ecological determinants, Working females

## Abstract

**Background:**

The majority of North American adult females do not meet current physical activity recommendations (150 min of moderate-to-vigorous intensity physical activity (MVPA) per week accrued in ≥10 min bouts) ultimately placing themselves at increased risk of morbidity and mortality. Working-age females face particular challenges in meeting physical activity recommendations as they have multiple demands, including occupational, family and social demands. To develop effective interventions to increase MVPA among working-age females, it is necessary to identify and understand the strongest modifiable determinants influencing these behaviours. Therefore, the objective of this systematic review is to examine the available evidence to identify intrapersonal, social and environmental determinants of MVPA among working-age females.

**Methods/Design:**

Six electronic databases will be searched to identify all prospective cohort studies that report on intrapersonal, social and/or environmental determinants of MVPA in working-age females. Grey literature sources including theses, published conference abstracts and websites from relevant organizations will also be included. Articles that report on intrapersonal (e.g. health status, self-efficacy, socio-economic status (SES), stress, depression), social environmental (e.g. crime, safety, area SES, social support, climate and capital, policies), and environmental (e.g. weather, workplace, home, neighbourhood, recreation environment, active transportation) determinants of MVPA in a working-age (mean age 18–65 years) female population will be included. Risk of bias will be assessed within and across all included studies using the Tool to Assess Risk of Bias in Cohort Studies and the Grades of Recommendation, Assessment, Development and Evaluation approach. Harvest plots will be used to synthesize results across all determinants, and meta-analyses will be conducted where possible among studies with sufficient homogeneity.

**Discussion:**

This review will provide a comprehensive examination of evidence in this field and will serve to highlight gaps for future research on the determinants of MVPA in working-age females and ultimately inform intervention design.

**Systematic review registration:**

PROSPERO: CRD42014009750.

## Background

In North America, only 3% to 14% of adult females [1,2] achieve at least 150 min (in bouts of 10 min or more) of moderate-to-vigorous intensity physical activity (MVPA) per week as recommended by the World Health Organization [3]. Research consistently shows that greater amounts and higher intensities of MVPA are protective against weight gain, chronic diseases including hypertension, heart disease, type 2 diabetes, colon cancer, breast cancer, and osteoporosis, and premature mortality [4-6].

The majority of adults spend a large portion of their days at work, limiting the amount of free time available for engaging in leisure pursuits such as physical activity [7,8]. Free time refers to the time in a day that remains after work and other necessary daily activities that have been performed and provides an important opportunity for rest, social interactions, leisure pursuits, and self-reflection [9]. Most working-age females generally report having even less free time than males, making it difficult for them to achieve the recommended levels of MVPA [8-10]. These females have to overcome several factors that compete for their time on a daily basis including occupational, family and social demands [10,11]. Working females have consistently reported difficulty in engaging in MVPA due to a variety of these factors [11]. Interestingly, employed females are more likely than males to report work demands as a barrier to their physical activity [12]. Females with children are also less physically active than females without children, and those with young children (<6 years) are less active than males with children the same age [13]. Females are also less active than their male peers [14,15] and are more likely to become inactive if they get married, have children or transition to paid employment [16-18].

**Figure 1:**
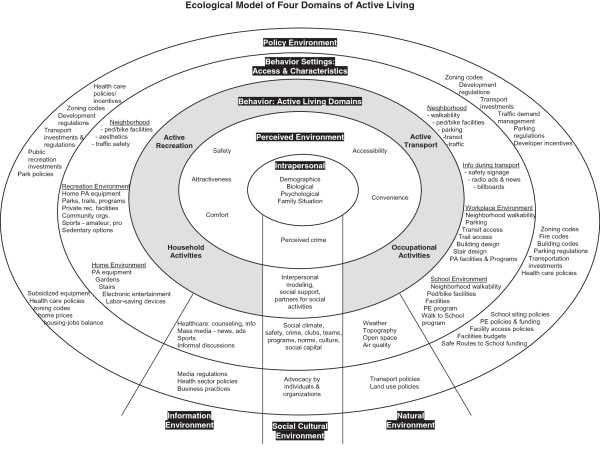
**Ecologic model of the four domains of active living/MVPA**[24]**(reproduced with permission from the author and publisher).**

Research examining the determinants of adult (both male and female) physical activity has widely applied socio-ecology theory as a framework [19-22]. The socio-ecology theory recognizes that individual behaviours are likely dependent on the dynamic relationships between multiple determinants (i.e. biology, motivation, self-efficacy, socio-cultural, policy, built and natural environments) across several levels (i.e. intrapersonal, interpersonal, workplace, community) [23]. Sallis et al. proposed an ecological model of MVPA as a conceptual approach to understanding the determinants of time spent being physically active within different domains (i.e. recreation, transport, household, and occupation) (Figure 1) [24]. Their model provides a schematic framework that recognizes the possible behaviour settings and contextual factors which have the capacity to influence time spent being physically active in these specific domains.

To develop effective interventions and appropriate health policies, it is necessary to identify and understand the potentially modifiable factors that influence a female’s propensity to be physically active and achieve the recommended 150 min of MVPA per week [3]. Previous reviews, though limited in number and now out-dated, examined the literature for factors related to physical activity uptake and adherence among females [25,26]. Importantly, none to date has specifically focused on working females who are likely to present with unique challenges in achieving work-life balance. Consequently, there is a need to identify which of these factors most strongly predict working-age females’ total, as well as domain-specific (work, home, leisure, transport) MVPA.

In addition to the growing body of evidence around determinants of MVPA, numerous interventions have been designed and tested to increase MVPA specifically among females, though fewer among working-age females, specifically [27-30]. Given that female adults spend the majority of their day at work and report limited free time, workplaces represent important settings for physical activity interventions. Although interventions are being proposed and tested [31,32], there has been relatively little work examining and synthesizing the available evidence to identify the strongest determinants of domain specific MVPA and/or total time spent in MVPA among working-age females, which may represent the best targets for intervention in this population. It is, therefore, imperative that the most important modifiable factors of MVPA among working-age females be identified for the development of effective interventions and appropriate health policies. In response to this need, the objective of the proposed systematic review is to identify intrapersonal, social and environmental determinants of domain-specific (work, home, transport, leisure) and total time spent in MVPA among working-age females.

## Methods/design

### Study design

A systematic review and meta-analysis will be undertaken to identify common and important intrapersonal, social and environmental determinants of MVPA in working-age females in high-income countries. The systematic review will adhere to the reporting guidelines of the *Preferred Reporting Items for Systematic Reviews and Meta-Analyses* (PRISMA) statement [33] and will meet the items outlined in *A Measurement Tool to Assess Systematic Reviews* (AMSTAR) checklist [34,35].

### Study registration

This systematic review has been prospectively registered with PROSPERO (registration number CRD42014009750; http://www.crd.york.ac.uk/PROSPERO).

### Criteria for considering studies for this review

#### Types of participants

Studies will be included if the sample is largely comprised of working-age females (≥80% or where female data can be separated), with a mean age between 18 and 65 years. Further, only studies from high-income countries will be included (those with a gross national income (GNI) per capita of $12,746 or more in 2013) [36]. Participant characteristics will be extracted for planned sub-group analyses (e.g. younger versus older, special populations such as types of occupation groups, work status (part-time, full-time, casual), women with children versus those without, and disease status). Studies with a mean age lower than 18 years or greater than 65 years or use of non-human participants (e.g. rats) will be excluded.

#### Types of exposures

Using an ecological model of active living [24], the review will examine intrapersonal (e.g. health status, self-efficacy, working status, socio-economic status, family status), social environmental (e.g. crime, safety, social support, social climate, social capital, policies), and physical environmental including natural environment and behaviour settings (e.g. weather, workplace environment, home environment, neighbourhood design, recreation environment, active transportation) determinants of MVPA among working-age females. The ecological model of active living recognizes that domain-specific physical activities are largely influenced by factors in the behaviour settings themselves, including the neighbourhood, recreation, home, workplace, school and transportation environments. Further, the social-cultural environment, policy environment, information environment and natural environments are recognized as playing a role within each of these domains and settings. The exposures will be identified as either objectively measured (e.g. crime rate in a neighbourhood) or perceived factors (e.g. feelings of safety in a neighbourhood). Studies where individual determinants cannot be isolated such as in the case of composite questionnaire scales will be excluded.

#### Types of comparators

Comparator or control groups are not applicable in this research as only prospective cohort studies are eligible.

#### Types of outcomes

As the main outcome of interest is MVPA level, studies that do not report on this will be excluded. MVPA is defined as a behaviour with an energy expenditure ≥3 metabolic equivalents (METs) $\left(1\;\mathrm{METs}=1\left(\frac{\mathrm{kcal}}{\mathrm{kg}*h}\right)\right)$, ≥40% of VO_2_ reserve, ≥45% peak VO_2_, ≥55% of peak heart rate, ≥12 on the Ratings of Perceived Exhaustion Scale (RPEs), or >100 steps per min [37-40]. For example, MVPA can be achieved by walking more than 3.2 km/h, cleaning (vacuuming, washing a car), or bicycling for pleasure [38]. Measures of association and risk between an exposure and MVPA will be captured from all studies. Depending on the number of studies identified for specific domains of MVPA, the review may be divided into multiple papers to better analyse determinants relevant to specific domains. Measures of time spent in total or domain-specific MVPA (e.g. min per day of MVPA from active recreation, transportation, household chores or occupation) or intensity (i.e. moderate and/or vigorous intensity physical activity) and where possible, a measure of variance around the outcome (e.g. standard error, 95% confidence intervals) will be extracted from all included studies regardless of the unit of measurement or method of measurement. MVPA can be either objectively measured (e.g. indirect calorimetry, accelerometers, activity monitors, observed patterns) or self-reported (e.g. physical activity questionnaire, journal or log). Potential and known health sequelae of MVPA (e.g. obesity, diabetes) are not of interest.

#### Type of studies

To obtain results describing determinants of MVPA, the systematic review will include all published and unpublished prospective cohort studies that quantify the association between a risk factor/determinant and the level of MVPA in working-age females. Although no language restrictions will be imposed in the search, only articles published in English or French will be included. A summary of this evidence and the confidence in this evidence will be conducted using Cochrane’s Grades of Recommendation, Assessment, Development and Evaluation (GRADE) approach [41] to increase internal validity of the review.

### Search methods for the identification of studies

A sensitive and comprehensive search strategy has been designed in collaboration with a research librarian (EW) and includes a search of six electronic databases: Ovid MEDLINE(R) In-Process (1946 to present); EBM Reviews—Cochrane Central Register of Controlled Trials (present); Embase Classic + (1947 to present); Ovid PsycINFO (1806 to present); SPORTDiscus (1830 to present); and dissertations and theses (1861 to present). The strategy is illustrated using the MEDLINE search as an example (Table 1) and will be modified according to the indexing systems of the other databases. Grey literature (non-peer reviewed works) that meets the inclusion criteria will be obtained including the following: published conference abstracts indexed under the bibliographic databases, published lists of theses and dissertations, government reports, and unpublished data and manuscripts (provided by original authors). Government reports will be searched using the Google search engine using a combination of key text words. The Google search engine will be used to identify studies that are published in non-indexed journals. Unpublished data and manuscripts will be solicited from authors of studies that report collecting MVPA, but in which, this data is not available within a published manuscript. Knowledgeable researchers in the field including those affiliated with the International Physical Activity and the Environment Network (http://www.ipenproject.org) will be solicited to identify other studies of interest. Finally, the bibliographies of all studies selected for the review will also be examined to identify further studies, as well as those of previous reviews.

**Table 1:** Sample MEDLINE search strategy

	**Search terms**
Outcome terms	
1	Exp exercise/
2	(moderate adj2 vigorous).tw.
3	MVPA.tw.
4	Physical* activit*.tw.
5	Exp sports/
6	Dancing/
7	Motor activity/
8	Physical fitness/
9	Aerobics.tw.
10	((moderate or vigorous or aerobic) adj2 exercise*).tw.
11	((moderate or vigorous or aerobic) adj2 activit*).tw.
12	Running.tw.
13	(cycling or biking).tw.
14	Swimming.tw.
15	Walking.tw.
16	(physical* adj (fit* or train* or exercise*)).tw.
17	“high intensit*” adj2 (exercise* or activit*).tw.
18	or/1-17
Physical environment terms	
19	Environment design/
20	Residence characteristics/
21	Poverty areas/
22	Built environment*.tw.
23	(walkable or walkability).tw.
24	(active adj (travel* or transportation or commut*)).tw.
25	((walking or pedestrian or cycling or bicycle or bike) adj (trail* or path* or route* or lane* or infrastructure)).tw.
26	((road or street) adj connectivity).tw.
27	(community adj2 (feature* or characteristic*)).tw.
28	Community design.tw.
29	Neighbo?rhood*.tw.
30	Sidewalk*.tw.
31	Green space.tw.
32	Parks.tw.
33	Public facilities/
34	Fitness centers/
35	((sport* or recreation* or exercise) adj facilit*).tw.
36	(“land use” adj2 mix*).tw.
37	(environment* adj (factor* or correlate* or determinant*)).tw.
38	Weather/
39	Weather.tw.
40	(gym or gyms).tw.
41	((fitness or recreation*) adj (centre* or center*)).tw.
42	or/19-41
Social environment terms	
43	Social environment/
44	Community networks/
45	Crime/
46	((safe* or unsafe) adj2 neighbo?rhood*).tw.
47	Social support/
48	Exp socioeconomic factors/
49	Culture/
50	Cultural characteristics/
51	(social* adj (capital or support* or influence* or environment* or connect* or correlate* or factor*)).tw.
52	(socioeconomic or socio-economic).tw.
53	(sociodemographic* or socio-demographic*).tw.
54	(cultural adj (factor* or correlate* or influence*)).tw.
55	Exp socioeconomic factors/
56	Public policy/
57	Health policy/
58	or/43-57
Intrapersonal terms	
59	Self efficacy/
60	Motivation/
61	Health status/
62	Attitude to health/
63	Health knowledge, attitudes, practice/
64	Health behaviour/
65	Self efficacy.tw.
66	Motivation.tw.
67	or/59-66
68	42 or 58 or 67
69	18 and 68
Population and study design limits	
70	Child/not exp adult/
71	Adolescent/not exp adult/
72	70 not 71
73	73 not 72
74	Exp aged/not adult/
75	74 not 75
76	Wom?n*.tw.
77	Exp women/
78	Female*.tw.
79	Female/
80	Womens health/
81	or/77-81
82	76 and 82
83	Exp animals/not humans/
84	83 not 84
85	Epidemiologic studies/
86	Exp cohort studies/
87	(follow up adj (study or studies)).tw.
88	(observational adj (study or studies)).tw.
89	Longitudinal.tw.
90	Cohort analy$.tw.
91	(cohort adj (study or studies)).tw.
92	Or/70-91
93	69 and 92

### Selection of studies

Citations retrieved from the search will be imported into EndNote X7 (Thompson Reuters, San Francisco, CA, USA), and duplicates will be removed using the ‘duplicate’ function. Remaining duplicates will be removed manually. Study eligibility assessment will be done independently in two stages by two reviewers (SAP, JLR). In the first stage, the reviewers will independently screen the titles and abstracts of all studies to identify eligible abstracts. In the second stage, the full texts of all abstracts that met the inclusion criteria or did not have insufficient information to judge eligibility in the abstract will be obtained and reviewed. If disagreements between the reviewers occur, consensus will be achieved through discussion and/or with the assistance of a third reviewer (EAK or RDR). Agreement will be measured at each stage. Reviewers will not be blinded to the authors or journals when screening articles.

### Data collection

Prior to data extraction, a standardized data extraction form will be created and pilot tested by the research team using a subset of the included studies. The extraction form will be modified based on feedback from the extractors to improve its usability and ensure that complete and appropriate information is obtained. Standardized data abstraction forms including quality assessments will be completed by one reviewer (SAP) and verified by another (JLR). If disagreements occur, consensus will be achieved through discussion and/or with assistant of a third reviewer (EAK or RDR). Reviewers will not be blinded to the authors or journals when extracting data.

From each included study, the following information will be extracted: lead author; year of publication; country of study; participant characteristics (age range and mean, sex distribution, health status, study setting); sample size; study design; length of follow-up (if applicable); exposure/determinant (separate entry for each determinant examined); measurement method for each exposure/determinant (including whether it is self-reported or objectively measured); level of the determinant (e.g. individual, social environment, physical environment); whether MVPA is self-reported or objectively measured; whether MVPA is reported as a daily total or under a specific domain; measurement method and units of measurement of MVPA; analytical methods used (e.g. unadjusted, adjusted/multivariate regression); relationship between the exposure/determinant and MVPA (significant positive, negative or absence of association; and effect on MVPA (e.g. increase, no change, or decrease in MVPA). Authors of suspected duplicate reports (i.e. reports on the same population and relationships between determinant and MVPA) will be contacted and in cases where several publications report the same results from the same data source, only one study per data source/analysis will be retained to avoid double counting.

If a paper employs a measure that has the potential to capture MVPA (e.g. International Physical Activity Questionnaire, accelerometers, etc.) but does not report on these outcomes in the manuscript or if a paper describes a study protocol related to MVPA, the authors will be contacted to ascertain whether the MVPA results can be provided. A maximum of three e-mail attempts will be made to contact the lead author of these studies to obtain additional information.

### Risk of bias within studies

Risk of bias will be assessed using the Tool to Assess Risk of Bias in Cohort Studies [42]. The tool includes an assessment of the following: selection bias (e.g. whether the exposed and unexposed groups were drawn from same population); performance bias (e.g. whether any co-interventions were present and if they influenced the exposed and unexposed similarly, confidence that the outcome of interest was not present at the start of the study); attrition bias (e.g. were the lengths of follow-up similar between exposed and unexposed); detection bias (e.g. confidence in the assessment of the exposures, outcomes and confounders, whether the study matched the exposed and unexposed groups for all variables associated with the outcome or was the analysis adjusted for these variables); and reporting bias (e.g. whether the outcomes were part of an *a priori* plan, whether the assessment of data could be reproduced). The risk of bias assessment will be carried out by two independent assessors (SAP and JLR), and if disagreements between assessors occur, consensus will be achieved through discussion with a third reviewer (RDR).

### Quality of the evidence

The quality of the evidence for the relationship between each exposure and MVPA will be assessed as high, moderate, low or very low using Cochrane’s GRADE approach [41]. Within this approach, non-randomized studies begin as low-quality evidence. In addition to study design, the quality of evidence will be rated upon possible risk of bias, imprecision, heterogeneity, indirectness, or suspicion of publication bias. Quality will be rated up if the summary of effect is large. GRADE suggests ‘considering rating up quality of evidence one level when methodologically rigorous observational studies show at least a two-fold reduction or increase in risk, and rating up two levels for at least a five-fold reduction or increase in risk’ [43]. GRADE Pro version 3.6.1 (GRADE Working Group) will be used to rate the quality of the evidence using the GRADE methodology.

### Planned analyses

Summary tables will be created to describe the populations, interventions (if applicable) and outcomes of all studies. The relationships between a large number of exposures and different levels (i.e. individual, neighbourhood) with MVPA will be assessed using a variety of methods. Due to the variety in the exposures and metrics used in the studies, the review will use Harvest plots [44,45] as a primary method of synthesis. Harvest plots allow results of the primary studies to be displayed across the various exposures and metric (e.g. perceived or objectively measured neighbourhood walkability) across various levels (i.e. individual, social environment, built environment) and across various outcome measurement methods (i.e. self-reported vs. objectively measured MVPA) to incorporate the strength of association, sample size, and study quality [44,45]. The Harvest plots will provide a graphical method to allow for a complete synthesis of the evidence and allow a comparison of the evidence across the various exposures.

Forest plots and meta-analyses will be created using Review Manager (RevMan) 5.3.3 (The Nordic Cochrane Centre, The Cochrane Collaboration, 2012) to synthesize the measures of effect (e.g. odds ratio, relative risk) and 95% prediction intervals for each exposure on MVPA. A random effects meta-analysis will be used as populations and modifying factors are likely to be similar but not identical across all studies. Heterogeneity will be assessed using the *I*^2^ statistic with values above 75% used to indicate high heterogeneity across studies [46]. Publication bias will be assessed using a funnel plot of the included studies’ estimates of effect. The plots will be assessed both visually and by using Egger’s test; with *p* <0.10 used to indicate the presence of a significant publication bias [47].

### Subgroup analyses

In addition to the primary analyses proposed, several *a priori* determined subgroup analyses will be performed when sufficient data are available. The analyses will examine differences between the following: age groups (e.g. 18–24 years versus 25–44 years versus 45–65 years); populations (e.g. healthy versus women with specific chronic diseases, between country groups i.e. North America versus Europe); occupation types (e.g. sedentary versus active); working status (i.e. full time, part time, homemaker, unemployed); self-reported and objectively measured exposures; self-reported and objectively measured MVPA; studies with high and low risk of bias and quality; different lengths of follow-up (e.g. <3 months, 3–6 months, >6 months); trends over time (e.g. by decade 1970s, 1980s, 1990s, 2000s, 2010s); and publication status (unpublished versus published results).

## Discussion

This systematic review will be the first, to our knowledge, to critically examine and synthesize the available literature assessing the relationships between intrapersonal, social and environmental determinants of total and domain-specific MVPA among working-age females. The review will provide a comprehensive examination of the evidence in the field to date and will serve to highlight gaps where future research on the determinants of working-age female MVPA remains to be conducted. The use of the Harvest plots will allow researchers to visually examine all of the determinants across the multiple levels of influence that are related to MVPA including the strength and quality of the evidence. It is anticipated that this review will be useful for a variety of stakeholders including those looking to design interventions targeting the most important modifiable factors to increase MVPA among this high-risk female population.

Recommendations from previous reviews included that interventions should be multifaceted, targeting a combination of factors including psychosocial, social environmental, workplace setting, community environment and public policy, as well as be designed with recognition of the multiple roles of females and integrating physical activity into daily routines [25,26].

## Abbreviations

AMSTAR: a measurement tool to assess systematic reviews; GRADE: Grades of Recommendation, Assessment, Development and Evaluation; METS: metabolic equivalents; MVPA: moderate-to-vigorous intensity physical activity; PRISMA: Preferred Reporting Items for Systematic Reviews and Meta-analyses; RPE: rating of perceived exertion.

## Competing interests

The authors declare that they have no competing interests.

## Authors’ contributions

SAP conceived the study, design and methodology, provided input for the bibliographic search strategy, and drafted and edited the manuscript. JLR also conceived of the study, its design and coordination and provided critical revision of the manuscript. EW conceived the bibliographic search strategy, participated in its design and provided critical revision of the manuscript. JGF, SH, EAK, KAM, KAN, and HET participated in the design of the study, provided expert input, and critically reviewed the manuscript. RDR also conceived of the study, provided methodological input, and critically reviewed the manuscript. All authors read and approved the final manuscript.
